# Isoliquiritigenin Suppresses Oral Squamous Cell Carcinoma Progression by Targeting FABP5-Mediated Lipid Metabolism: Association with the circPOLB/miR-548ae-3p/C-MYC Axis

**DOI:** 10.32604/or.2026.081109

**Published:** 2026-07-16

**Authors:** Liang Li, Hong Deng, Zhiyong Li, Yu Li, Lingrui Liu, Lan Xie, Yue Chen

**Affiliations:** 1 Department of Stomatology, the First Affiliated Hospital of Guangdong Pharmaceutical University, Guangzhou, China; 2 State Key Laboratory of Oncology in South China, Guangdong Provincial Clinical Research Center for Cancer, Sun Yat-Sen University Cancer Center, Guangzhou, China; 3 Department of Radiation Oncology, Nanfang Hospital, Southern Medical University, Guangzhou, China

**Keywords:** Oral squamous cell carcinoma, miR-548ae-3p, c-MYC, circPOLB, FABP5

## Abstract

**Objectives:** Oral squamous cell carcinoma (OSCC) is a common and deadly cancer affecting the oral cavity. This study aims to explore the regulatory role and molecular mechanism of miR-548ae-3p in OSCC proliferation, invasion, and lipid metabolism, as well as the therapeutic potential of isoliquiritigenin (ISL) targeting OSCC lipid metabolism. **Methods:** Expression levels of miR-548ae-3p were measured in OSCC cell lines and normal oral keratinocytes using real-time quantitative polymerase chain reaction. Functional assays, such as cell counting Kit-8 proliferation and Transwell invasion assays, evaluated the effects of miR-548ae-3p overexpression in CAL-27 and SCC-25 cells. Bioinformatic prediction and dual-luciferase reporter assays investigated interactions among miR-548ae-3p, hsa_circRNA_0001794 (circPOLB), and cellular myelocytomatosis oncogene (c-MYC). Lipid metabolism was assessed using lipid droplet staining, fatty acid oxidation assays, total fatty acids and palmitic acid quantification, and fatty acid-binding protein 5 (FABP5) expression analysis. The inhibitory effects of ISL on OSCC lipid metabolism and invasiveness were also examined. **Results:** MiR-548ae-3p was downregulated in OSCC cells compared to normal keratinocytes (n = 3, *p* < 0.001). miR-548ae-3p overexpression inhibited the proliferation and invasion of CAL-27 and SCC-25 cells (n = 3, *p* < 0.001). CircPOLB functions as a molecular sponge for miR-548ae-3p, which in turn targets c-MYC, a key oncogene. MiR-548ae-3p overexpression reduced lipid droplet accumulation, fatty acid oxidation, total fatty acid content, and intracellular palmitic acid levels, accompanied by downregulation of FABP5 (n = 3, *p* < 0.001). Furthermore, ISL treatment decreased FABP5 expression, fatty acid metabolism, and invasive capacity of OSCC cells (n = 3, *p* < 0.001), supporting its potential as a therapeutic agent. **Conclusions:** MiR-548ae-3p displays tumor-suppressive activity in OSCC, restraining proliferation, invasion, and fatty-acid metabolism through engagement of the circPOLB/c-MYC axis and is associated with reduced FABP5 expression. Targeting lipid metabolism using agents like ISL could be a promising approach for treating OSCC.

## 1 Introduction

Oral squamous cell carcinoma (OSCC) primarily develops in the oral mucosa and lips, representing 90–95% of malignant tumors in these regions [1,2]. The incidence of OSCC exhibits distinct geographic variations and is strongly associated with multiple risk factors [3]. Human papillomavirus (HPV) infection is the main risk factor for OSCC in young populations [4,5]. Recent advances in the treatment of OSCC have centered on a multidisciplinary approach combining surgery, radiotherapy, chemotherapy, and targeted biological therapies, such as epidermal growth factor receptor (EGFR) inhibitors [6,7]. Despite progress, treatment outcomes remain highly dependent on the tumor’s initial stage and grade [8,9]. Moreover, the adverse effects of surgery and chemoradiotherapy—such as impairment of swallowing, mastication, and speech functions—significantly diminish patients’ quality of life [10]. Neoadjuvant chemotherapy shows limited efficacy in locally advanced OSCC, contributing to the persistently high mortality rates [11]. These challenges underscore the urgent need for additional investigations into the mechanisms driving OSCC and the identification of novel therapeutic targets to enhance diagnosis, treatment strategies, and patient prognosis.

Tumorigenesis is fundamentally a genetic disease; however, gene mutations alone do not fully account for the complex processes of tumor growth and metastasis [12,13]. Recent research on non-coding RNAs highlights the crucial roles of microRNAs (miRNAs) in cancer development and progression [14,15,16]. Evidence shows that miRNAs regulate key tumor cell behaviors—such as proliferation, apoptosis, invasion, and migration—mainly by suppressing the transcription of their target genes [17,18]. miRNAs of the miR-548 family exhibit dual roles in cancer, contingent on the specific cancer type and molecular context. For instance, Chen et al. [19] reported that miR-548 is upregulated in gastric cancer and functions as an oncogene and promotes invasion, correlating with poor patient prognosis. Besides, studies in breast cancer have demonstrated that miR-548 members inhibit multidrug resistance and cell survival pathways. Saberiyan et al. [20] revealed that miR-548k regulates the expression of the ABCG2 transporter, a key mediator of chemoresistance, while Yadollahi-Farsani et al. [21] demonstrated that miR-548k inhibits apoptosis by targeting the PTEN/PI3K/AKT signaling pathway, thereby promoting breast cancer progression.miR-548ac acts as a tumor suppressor by promoting apoptosis through the downregulation of TMEM158 in laryngeal squamous cell carcinoma [22].

Beyond their canonical roles in proliferation and invasion, miRNAs have emerged as important post-transcriptional regulators of cancer lipid metabolism, acting on key lipogenic transcription factors and fatty-acid–handling enzymes; for example, miR-185 and miR-342 directly target SREBP-1/2 to suppress FASN- and HMGCR-driven lipogenesis in prostate cancer [23], and broader reviews have catalogued miRNAs that modulate fatty-acid synthesis, β-oxidation, and uptake across tumour types [24]. Independently, c-MYC has been established as a master regulator of cancer lipid metabolism, directly upregulating fatty-acid transporters, elongases, and oxidation programmes in breast and pancreatic cancers [25,26]. Several miRNAs—most notably miR-34a—exert tumour-suppressive activity by directly targeting the c-MYC 3′ UTR [27], providing a precedent for the broader concept that miRNA–MYC interactions can rewire downstream metabolic programmes. These converging observations motivated us to ask whether miR-548ae-3p, through engagement of c-MYC, contributes to the lipid-metabolic phenotype of OSCC. miR-548ae-3p belongs to the miR-548 family; unlike other family members with established roles in breast or gastric cancers, the specific function of miR-548ae-3p in the oral cavity remains unexplored. Cui et al. indicate that hsa_circ_0006646 can counteract miR-548ae-3p’s inhibitory effect on MFAP2 by competitively binding to miR-548ae-3p, ultimately promoting the epithelial-mesenchymal transition (EMT) in esophageal cancer [28]. However, the involvement of miR-548ae-3p in OSCC is not well understood, especially in relation to lipid metabolism, which is crucial for cancer cell energy balance and invasiveness [29].

Given the emerging importance of altered lipid metabolism in OSCC pathogenesis, elucidating the regulatory mechanisms could reveal novel therapeutic targets. This study aims to explore the role of miR-548ae-3p in the metabolic reprogramming and malignant progression of OSCC.

## 2 Methods and Materials

### 2.1 Cell Culture and Treatment

Four authenticated human OSCC cell lines—SCC-9 (ATCC CRL-1629), SCC-15 (ATCC CRL-1623), SCC-25 (ATCC CRL-1628), and CAL-27 (ATCC CRL-2095)—were obtained from the ATCC (Manassas, VA, USA). Primary normal human oral keratinocytes (HOK), used as the non-malignant control, were obtained from ScienCell Research Laboratories (Cat# 2610, Carlsbad, CA, USA). All cell lines were cultured according to the suppliers’ recommendations. All cell lines were authenticated by STR profiling and routinely confirmed mycoplasma-negative. Gene modulation in SCC-25 and CAL-27 cells was achieved by transfecting miRNA mimics, inhibitors, and siRNAs from GenePharma (Shanghai, China; Table 1) and transfected into SCC-25 and CAL-27 cells using Lipofectamine 3000 (Cat# L3000008, Thermo Fisher Scientific, Waltham, MA, USA). SCC-25 and CAL-27 cells were treated with 60 μmol/L isoliquiritigenin (ISL, Cat# I3766, Sigma-Aldrich, St. Louis, MO, USA) dissolved in dimethyl sulfoxide (DMSO) for 24 h, with DMSO-treated cells serving as vehicle controls. Treatment durations and concentrations were optimized based on a previous study [30]. The mature miRNA sequence of hsa-miR-548ae-3p mimic used in this study: CAAAAACUGCAAUUACUUUCA; Stem-loop Sequence: GCAGUUUUUGCCAUUAAGUUGCGGUUUUUGCCAUUAUAAUGGCAAAAACUGCAAUUACUUUCACACCUGC.

**Table 1:** The sequences of siRNAs used in this study.

siRNA	Species	Sequences
si-NC	Human	UUCUCCGAACGUGUCACGUTT
si-circPOLB	Human	GAGTGGAGCTGAAGCTAAGAATT

### 2.2 In Vitro Experiments

#### 2.2.1 Cellular Functional Assays

Cell proliferation was evaluated with the CCK-8 assay (GlpBio, Montclair, CA, USA) according to the manufacturer’s instructions. Cells were seeded into 96-well plates at 2000–3000 cells/well, cultured under the indicated treatments for the designated time intervals, and the absorbance at 450 nm was measured on a microplate reader. For the colony formation assay, cells were seeded into 6-well plates at 600 cells/well and subjected to the specified treatments or transfections; after 10–14 days, colonies were fixed with methanol, stained with 0.1% crystal violet, washed, air-dried, and counted under a microscope. Cell invasion was assessed using Matrigel-coated 24-well Transwell inserts (8 μm pore, Corning, Corning, NY, USA). Cells (5 × 10^4^) suspended in serum-free medium were added to the upper chamber, with medium containing 10% FBS in the lower chamber as a chemoattractant. After incubation for 24 h at 37°C, cells that had invaded the lower membrane surface were fixed with 4% paraformaldehyde, stained with crystal violet, and counted microscopically in five randomly selected fields [31].

#### 2.2.2 Dual-Luciferase Reporter Assay

The sequences of circPOLB and the c-MYC 3′ untranslated region (3′UTR), containing the predicted miR-548ae-3p binding sites, were inserted into the psiCHECK-2 vector, and site-directed mutagenesis was used to generate the corresponding mutant constructs. Cells were seeded into 12-well plates at ~50% confluence and, at ~70% confluence, co-transfected in serum-free medium with 50 ng of reporter plasmid per well together with either miR-548ae-3p mimics or mimic negative control (NC) at a final concentration of 20 nM, using 0.5 μL transfection reagent per well; each sample was assayed in triplicate. After 36–48 h, firefly and Renilla luciferase activities were measured using the Dual-Luciferase Reporter Assay Kit (Cat# E1910, Promega, Madison, WI, USA) on a microplate reader.

#### 2.2.3 Quantitative Real-Time PCR

RNA was extracted utilizing the RNA-Quick Purification Kit from YiShen Biotech (Shanghai, China). cDNA synthesis for circRNAs and mRNAs was performed using PrimeScript™ Master Mix (TaKaRa, Shiga, Japan). miRNA detection involved generating cDNA with the PrimeScript™ RT Reagent Kit, followed by quantification using the SYBR Green Master Mix, both from TaKaRa (Shiga, Japan). Reactions were run on a Bio-Rad CFX96 Touch™ Real-Time PCR Detection System (Bio-Rad, Hercules, CA, USA). GAPDH served as the endogenous control for circRNA/mRNA and U6 for miRNA. Relative expression was calculated using the 2^−ΔΔCt^ method. miR-548ae-3p was reverse-transcribed and quantified using commercial Bulge-Loop™ miRNA qRT-PCR primer sets (RiboBio, Guangzhou, China) according to the manufacturer’s instructions. The primer sequences are listed in Table 2.

**Table 2:** The primer sequences for qRT-PCRs used in this study.

Construct	Species	Direction	Sequence (5′-3′)
circPOLB	Human	Forward	AAAAGCAGCATCTGTTATAGCA
		Reverse	CTTTTCCAGTTTACGTAATTTT
FABP5	Human	Forward	GGTGCATTGGTTCAGCATCAGG
		Reverse	TCATAGATCCGAGTACAGGTGAC
GAPDH	Human	Forward	GGAGCGAGATCCCTCCAAAAT
		Reverse	GGCTGTTGTCATACTTCTCATGG
MYC	Human	Forward	CCTGGTGCTCCATGAGGAGAC
		Reverse	CAGACTCTGACCTTTTGCCAGG
U6	Human	Forward	CTCGCTTCGGCAGCACA
		Reverse	AACGCTTCACGAATTTGCGT

#### 2.2.4 Western Blot

Total protein was extracted in RIPA lysis buffer containing protease and phosphatase inhibitors, and protein concentrations were determined with a BCA protein assay. Equal amounts of protein were separated by SDS-PAGE and transferred to PVDF membranes. After blocking, membranes were incubated overnight at 4°C with primary antibodies against c-MYC (1:1000; Cat# 9402, Cell Signaling Technology, Danvers, MA, USA), FABP5 (1:1000; Cat# 39926, Cell Signaling Technology, Danvers, MA, USA), and GAPDH (1:8000; Cat# 10494-1-AP, Proteintech, Rosemont, IL, USA), followed by an HRP-conjugated secondary antibody (1:5000; Cat# bs-0295G, Bioss, Beijing, China). Immunoreactive bands were visualized with the NcmECL Ultra high-sensitivity ECL kit (Cat# P10100, NCM Biotech, Suzhou, China) and quantified by densitometry using ImageJ software, with GAPDH as the loading control.

#### 2.2.5 Oil Red O Staining

Cells were fixed with 4% paraformaldehyde, washed, treated with 60% isopropanol, and stained with freshly prepared Oil Red O working solution for 10–15 min at room temperature in the dark. After differentiation with 60% isopropanol and rinsing with distilled water, intracellular lipid droplets were imaged by light microscopy, with hematoxylin counterstaining of nuclei for qualitative assessment. For quantification, a parallel set of wells was processed without counterstaining: the retained Oil Red O was eluted with 100% isopropanol for 10 min at room temperature, and the absorbance of the eluate was measured at 520 nm with a spectrophotometer (Bio-Rad, Hercules, CA, USA). Results were expressed as relative lipid content after normalization to cell number.

### 2.3 Fatty Acid Metabolism Analysis

Fatty acid oxidation (FAO) was quantified by measuring ^14^CO_2_ production from [1-^14^C]-palmitic acid. Cells were incubated with 100 μM [1-^14^C]-palmitic acid complexed with fatty acid-free BSA (50 mg/mL) for 4 h at 37°C, and the released ^14^CO_2_ was trapped in 1 M KOH and measured by liquid scintillation counting (PerkinElmer Tri-Carb, Waltham, MA, USA); data were normalized to total protein. Total fatty acid content was determined by chloroform–methanol extraction, conversion to methyl esters, and gas chromatography–mass spectrometry (Agilent 7890B/5977B, Santa Clara, CA, USA) with heptadecanoic acid (C17:0) as internal standard. Intracellular palmitic acid was extracted similarly and quantified by liquid chromatography–mass spectrometry (Agilent 1290/6470, Santa Clara, CA, USA) against a palmitic acid standard curve. Palmitic acid was chosen as the representative species, being the primary substrate for mitochondrial β-oxidation.

### 2.4 Expression and Clinical Correlation Analysis

All analyses were based on the TCGA head and neck squamous cell carcinoma (TCGA-HNSC) cohort. Differential expression of c-MYC between HNSC tumor and normal tissues was analyzed using UALCAN (https://ualcan.path.uab.edu/analysis.html) [32] with significance assessed by the platform’s default Student’s *t*-test. The correlation between c-MYC and FABP5 was evaluated in HNSC tumor samples using the Correlation module of GEPIA2 (http://gepia2.cancer-pku.cn/) [33], with the Spearman coefficient computed on non-log-scale data and displayed on log-scale axes. The prognostic value of c-MYC was assessed with the Survival module of GEPIA2 (overall survival, TCGA-HNSC); patients were stratified into high- and low-expression groups by the median (50%) cutoff, hazard ratios were estimated by a Cox proportional-hazards model with 95% confidence intervals, and survival differences were evaluated by the Log-rank test (*p* < 0.05 considered significant).

### 2.5 Statistical Analysis

All experiments were performed using at least three independent biological replicates. For each biological replicate, the qRT-PCR, dual-luciferase reporter, CCK-8, FAO, total fatty acid, and palmitic acid assays were measured in three technical replicates and averaged. Data are presented as mean ± standard deviation (SD). Statistical analyses were performed using GraphPad Prism 10 (GraphPad Software, La Jolla, CA, USA). Before parametric testing, data were assessed for normality (Shapiro–Wilk test) and homogeneity of variance (Levene’s test). Comparisons between two groups used unpaired Student’s *t*-test; comparisons across more than two groups used one-way ANOVA followed by Dunnett’s post hoc test. A *p*-value below 0.05 was considered statistically significant.

## 3 Results

### 3.1 miR-548ae-3p Suppresses Proliferation and Invasion in Oral Squamous Cell Carcinoma

**Figure 1:**
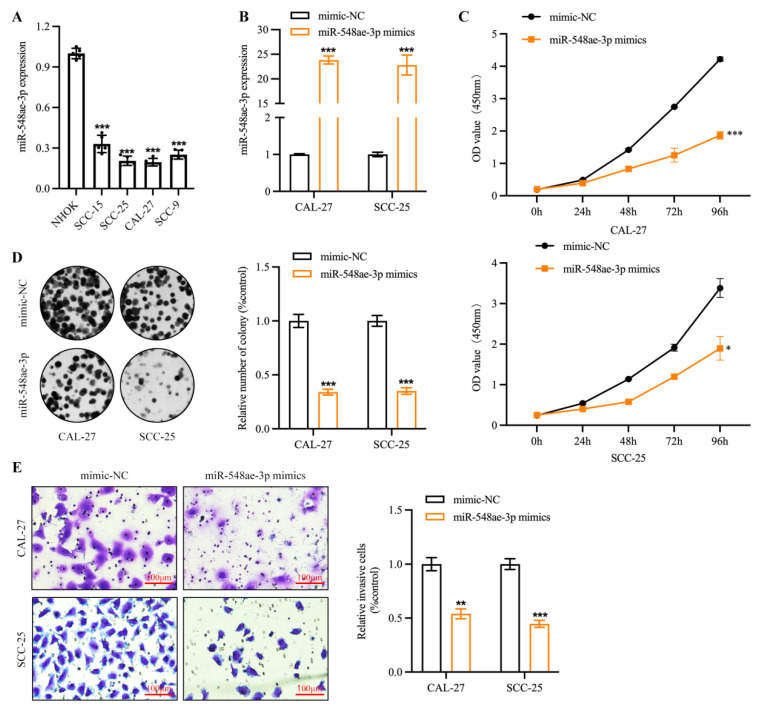
miR-548ae-3p Suppresses Proliferation and Invasion in OSCC. (**A**) RT-qPCR quantified miR-548ae-3p expression in different OSCC cell lines. (**B**) RT-qPCR served to confirm the efficiency of miR-548ae-3p overexpression. (**C**) CCK-8 assays were performed to evaluate the growth curves of CAL-27 and SCC-25 cells. (**D**) Colony formation assays evaluated the impact of miR-548ae-3p overexpression on the clonogenic potential of these cells. (**E**) Transwell invasion assays evaluated the impact of miR-548ae-3p on the invasiveness of these cells. *p < 0.05, **p < 0.01, ***p < 0.001.

miR-548ae-3p expression levels were assessed in OSCC cell lines. RT-qPCR analysis demonstrated that miR-548ae-3p was significantly downregulated in OSCC cell lines compared to NHOK (Fig. 1A). CAL-27 and SCC-25, which showed the lowest expression levels, were chosen for further miR-548ae-3p overexpression experiments (Fig. 1B). CCK-8 assays demonstrated that overexpressing miR-548ae-3p significantly inhibited cell proliferation compared to controls (Fig. 1C). Colony formation assays revealed that miR-548ae-3p overexpression inhibited the colony-forming ability of OSCC cells (Fig. 1D). Transwell assays demonstrated that miR-548ae-3p overexpression significantly decreased the invasiveness of OSCC cells (Fig. 1E).

### 3.2 circPOLB Promotes c-MYC Expression by Competitively Binding miR-548ae-3p

**Figure 2:**
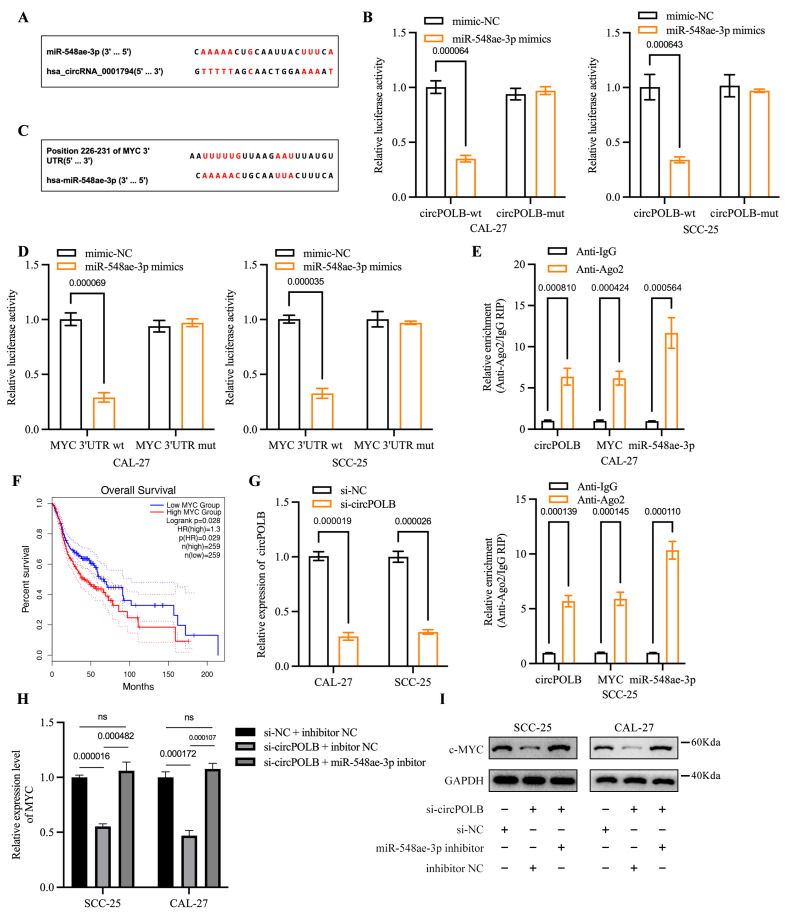
circPOLB Promotes c-MYC Expression by Competitively Binding miR-548ae-3p. (**A**) Predicted binding sites between miR-548ae-3p and circPOLB. (**B**) Dual-luciferase reporter assay comparing the effects of wild-type and mutant circPOLB constructs. (**C**) Predicted miR-548ae-3p binding sites located in the c-MYC 3′ untranslated region. (**D**) The luciferase activity was detected in the MYC-wt and MYC-mut group co-transfection with miR-548ae-3p mimics or negative control. (**E**) RNA immunoprecipitation (RIP) assay was performed to detect the relative enrichment of circPOLB, MYC, and miR-548ae-3p in the Ago2 complex. (**F**) Kaplan-Meier estimates of overall survival stratified by MYC expression. (**G**) RT-qPCR was used to confirm the efficiency of circPOLB knockdown. (**H**,**I**) Analysis of c-MYC mRNA (**H**) and protein (**I**) expression was conducted in CAL-27 and SCC-25 cells across three experimental conditions: the control group (si-NC + inhibitor NC), the circPOLB knockdown group (si-circPOLB + inhibitor NC), and the rescue group (si-circPOLB + miR-548ae-3p inhibitor). ns, not significant.

Given that miRNAs are often closely associated with circular RNAs (circRNAs) via the competing endogenous RNA (ceRNA) mechanism in tumor cells [15], we conducted an investigation into the circRNAs that regulate miR-548ae-3p. Bioinformatic analysis predicted that circPOLB harbors binding sites for miR-548ae-3p (Fig. 2A). CircPOLB, also known as hsa_circRNA_0001794 (chr8:42202470-42206608), originates from the exon region of the POLB gene located at 8p11.21. Dual-luciferase reporter assays showed that luciferase activity was higher in cells co-transfected with wild-type circPOLB (circPOLB-wt) and mimic negative control (mimic-NC) compared to those co-transfected with circPOLB-wt and miR-548ae-3p mimics. Conversely, cells transfected with mutant circPOLB (circPOLB-mut) showed no significant difference in luciferase activity regardless of mimic-NC or miR-548ae-3p mimics transfection (Fig. 2B). TargetScan analysis further identified c-MYC as a putative target of miR-548ae-3p (Fig. 2C). Further dual-luciferase reporter assays demonstrated that altering the miR-548ae-3p binding site in the c-MYC 3′ untranslated region abolished the luciferase activity differences between cells transfected with mimic-NC and those with miR-548ae-3p mimics (Fig. 2D). To further validate the interactions of circPOLB and MYC with miR-548ae-3p, Ago2-RIP assays were performed in CAL-27 and SCC-25 cells. The results showed that circPOLB, MYC, and miR-548ae-3p were predominantly enriched in the Ago2 complex (Fig. 2E). To evaluate the prognostic value of c-MYC, we stratified patients into high and low expression groups (n = 259 per group) and performed a Kaplan-Meier survival analysis (Fig. 2F). The results indicated that patients with high c-MYC expression exhibited significantly shorter overall survival compared to those in the low MYC group (Log-rank *p* = 0.028). To explore circPOLB’s role in regulating c-MYC expression via miR-548ae-3p sponging, CAL-27 and SCC-25 cells were transfected with si-NC and inhibitor-NC as controls, si-circPOLB to reduce circPOLB expression (Fig. 2G), and si-circPOLB combined with a miR-548ae-3p inhibitor for rescue analysis. Silencing circPOLB significantly reduced c-MYC expression, whereas inhibition of miR-548ae-3p restored c-MYC levels (Fig. 2H,I).

### 3.3 Overexpression of miR-548ae-3p Attenuates Fatty Acid Oxidation and Lipid Uptake in Oral Squamous Cell Carcinoma

**Figure 3:**
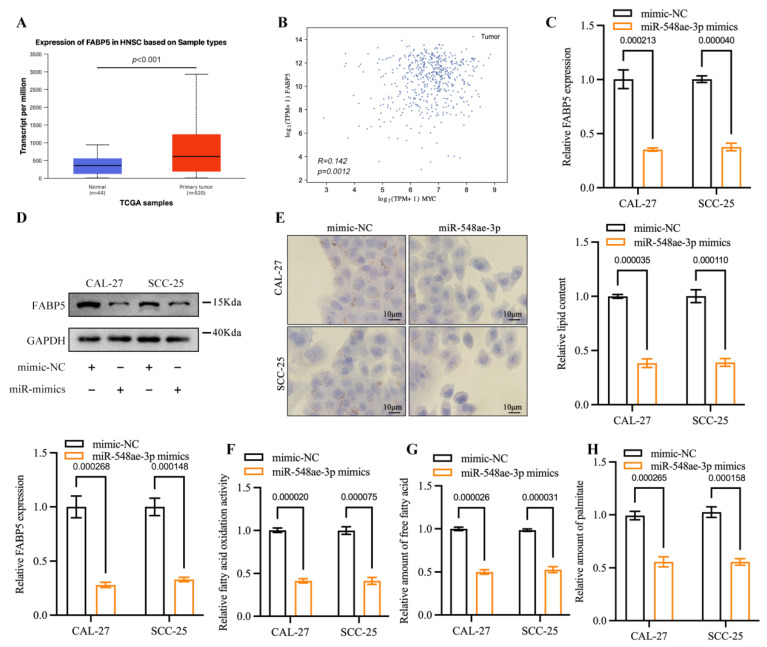
Overexpression of miR-548ae-3p attenuates fatty acid oxidation and lipid uptake in Oral Squamous Cell Carcinoma. (**A**) FABP5 mRNA expression levels in tumor tissues versus normal tissues from the TCGA-HNSC cohort. (**B**) Spearman correlation analysis between MYC and FABP5 mRNA expression in the TCGA-HNSC dataset (n = 520; *r* = 0.142, *p* = 0.0012). (**C**,**D**) qRT-PCR (**C**) and Western blot (**D**) analyses showing the mRNA and protein expression levels of FABP5 in CAL-27 and SCC-25 cells following transfection with miR-548ae-3p mimics. (**E**) Representative microscopic images of lipid droplet accumulation in OSCC cells. (**F**–**H**) Quantitative analyses of fatty acid oxidation (FAO) activity (**F**), total free fatty acid content (**G**), and palmitic acid levels (**H**) in CAL-27 and SCC-25 cells overexpressing miR-548ae-3p.

Building on our finding that c-MYC is a downstream effector of the circPOLB/miR-548ae-3p axis, we sought to elucidate the functional consequences of this regulation on OSCC metabolic reprogramming. We focused on fatty acid-binding protein 5 (FABP5), a key transporter in lipid metabolism. Analysis of the TCGA-HNSC cohort revealed that FABP5 was significantly overexpressed in tumor tissues compared to normal tissues (Fig. 3A). Crucially, a positive correlation was observed between c-MYC and FABP5 mRNA levels in this cohort (r = 0.142, *p* = 0.0012; Fig. 3B). To verify this regulatory link, we performed molecular and functional assays. Both qRT-PCR and Western blot analyses confirmed that miR-548ae-3p overexpression—which targets the c-MYC axis—markedly suppressed FABP5 expression at both mRNA and protein levels in CAL-27 and SCC-25 cells (Fig. 3C,D). Consequently, the impact of miR-548ae-3p on lipid metabolism was evaluated. Lipid metabolism assays demonstrated that miR-548ae-3p overexpression significantly reduced lipid droplet formation, decreased FAO pathway activity, and lowered overall fatty acid content (Fig. 3E–G). Furthermore, quantitative analysis revealed a substantial reduction in palmitic acid levels following miR-548ae-3p mimics transfection (Fig. 3H). Collectively, miR-548ae-3p overexpression reduced FABP5 expression and was accompanied by decreased lipid droplet accumulation, fatty-acid oxidation, total fatty acids, and intracellular palmitate in OSCC cells.

### 3.4 ISL Inhibits Oral Squamous Cell Carcinoma Invasion by Attenuating Fatty Acid Oxidation and Lipid Uptake

**Figure 4:**
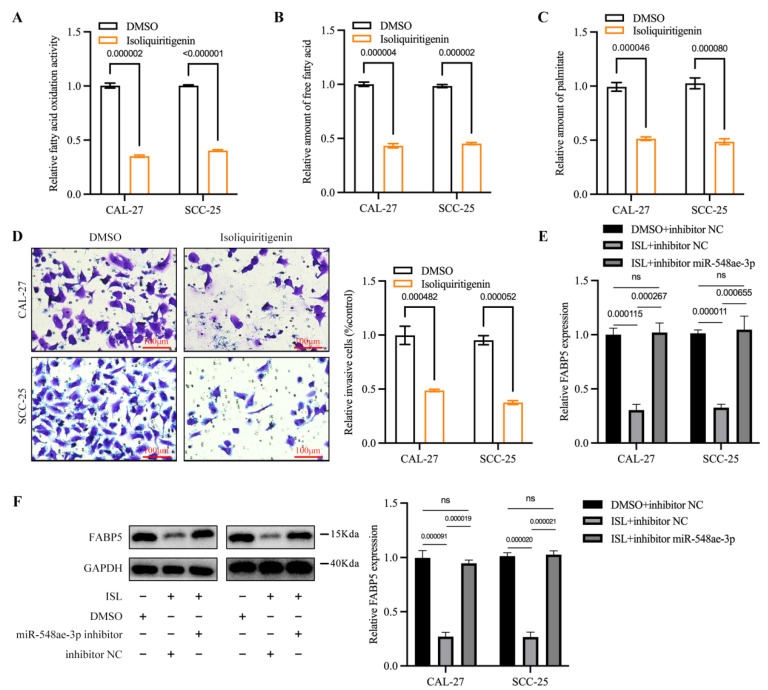
ISL Inhibits Oral Squamous Cell Carcinoma Invasion by Attenuating Fatty Acid Oxidation and Lipid Uptake. (**A**–**C**) Quantitative measurements assessing the influence of ISL on fatty acid oxidation activity, overall free fatty acid content, and palmitic acid concentration. (**D**) Transwell invasion assays measuring the effect of ISL on the invasive behavior of OSCC. (**E**,**F**) Analysis of FABP5 mRNA (**E**) and protein (**F**) expression under three experimental conditions: control group (DMSO + inhibitor NC), ISL treatment group (ISL + inhibitor NC), and rescue group (ISL + miR-548ae-3p inhibitor). ns, not significant.

To investigate the clinical potential of ISL in oral squamous cell carcinoma, we detected its impacts on lipid metabolism and invasion ability in OSCC cells. Quantitative analyses revealed that ISL reduced FAO (Fig. 4A), free fatty acid levels (Fig. 4B), and palmitic acid concentrations (Fig. 4C), demonstrating its inhibitory effect on lipid metabolism in these cancer cells. Additionally, functional assays confirmed that ISL diminished the invasive capacity of OSCC cells (Fig. 4D). To verify whether ISL regulates FABP5 expression through the above circPOLB/miR-548ae-3p/c-MYC axis, and further modulates OSCC cell lipid metabolism, we performed rescue experiments. The results showed that ISL treatment significantly downregulated FABP5 expression, while inhibition of miR-548ae-3p could restore its expression (Fig. 4E,F). Collectively, ISL treatment reduced FABP5 expression, fatty-acid oxidation, total fatty acids, palmitate, and invasion in OSCC cells, and the ISL-induced reduction of FABP5 was attenuated by co-treatment with a miR-548ae-3p inhibitor.

## 4 Discussion

Lipid metabolism is a pivotal aspect of metabolic reprogramming in cells [34]. Lipids not only serve as essential components of cellular membranes but also as key sources of ATP [35]. Moreover, they act as second messengers in intracellular signaling pathways. Research indicates that fatty acid metabolism in tumor cells differs markedly from that in normal cells [36,37]. Metabolic reprogramming, particularly enhanced FAO and uptake via FABPs, is a critical adaptation for tumor survival in hypoxic environments [38,39,40]. While c-MYC is a known regulator of these processes, the upstream epigenetic networks driving this axis in OSCC have remained elusive [34]. Building on these mechanisms, pharmacological agents targeting critical steps in fatty acid metabolism are currently being developed. Notably, FABP inhibitors have been shown to inhibit tumor progression by attenuating MYC protein activity, which leads to reduced tumor cell proliferation [41].

In this study, we advance this understanding by providing the first evidence that the circPOLB/miR-548ae-3p axis acts as a critical upstream epigenetic switch controlling this metabolic machinery in OSCC. The MYC gene, an established oncogene on chromosome 8, which also contains POLB, the parent gene of circPOLB, is crucial in cancer biology [42]. The MYC family, including c-MYC, n-MYC, and l-MYC, plays a crucial role in the onset and development of numerous cancers [43,44]. c-MYC alterations—including focal 8q24 amplification and transcriptional overexpression—are among the most recurrent oncogenic events in HPV-negative OSCC and are reported in a substantial proportion of clinical cases [45]. As a transcription factor, c-MYC regulates key tumor-related processes, including multidrug resistance, cell cycle progression, apoptosis, and lipid metabolism [46].

Building on the established role of MYC in OSCC, our study further elucidates the upstream regulatory mechanisms that modulate c-MYC expression and its downstream effects on tumor metabolism and progression. We identified circPOLB as a critical regulator that promotes OSCC malignancy through a competitive endogenous RNA mechanism. circPOLB acts as a molecular sponge for miR-548ae-3p, sequestering the microRNA and alleviating its suppression of c-MYC. This derepression results in the significant upregulation of c-MYC, which, in turn, enhances the expression of FABP5, a key mediator of fatty acid metabolism. Our findings regarding the tumor-suppressive role of miR-548ae-3p offer an important counterpoint to the dual nature observed in the miR-548 family [19]. While Chen et al. [19] reported that miR-548 acts as an oncogene in gastric cancer, our results align more closely with findings in breast and laryngeal cancers, where miR-548 members inhibit progression [20,22]. This result is consistent with the previous findings by Kawaguchi et al. Their study demonstrated that the overexpression of FABP5 in prostate cancer cells is associated with hypomethylation of CpG islands in the promoter region, and that c-MYC, as a direct trans-acting factor, can upregulate FABP5 expression [47]. However, the upstream epigenetic or non-coding RNA regulatory network controlling this axis in OSCC is unknown. Our study fills this mechanistic gap by identifying the circPOLB/miR-548ae-3p axis as a novel upstream switch that dictates c-MYC/FABP5 activity. By linking non-coding RNA dysregulation directly to metabolic reprogramming. Crucially, the interpretation of these mechanisms depends on the genetic context of the models used. We utilized CAL-27 and SCC-25 cell lines, which are characterized as HPV-negative and c-MYC amplified [45,48]. This distinction is vital because OSCC exhibits significant metabolic heterogeneity. HPV-negative tumors typically rely on host genetic drivers (e.g., MYC amplification) for metabolic reprogramming, whereas HPV-positive tumors display distinct viral-driven profiles [48]. Therefore, the circPOLB/c-MYC/FABP5 axis likely represents a primary metabolic dependency specifically in the HPV-negative/c-MYC-high subtype of OSCC. More broadly, the circPOLB/miR-548ae-3p axis described here sits within an expanding landscape of non-coding RNA networks that regulate intracellular signalling and intercellular communication relevant to metastatic behaviour and therapeutic responsiveness across tumour types [49] suggesting that mechanistic principles uncovered in OSCC may have parallels in other malignancies.

Therapeutically, we demonstrate that IS suppresses this axis and inhibits invasion. The circPOLB/miR-548ae-3p/c-MYC signaling axis emerges as an important pathway driving metabolic reprogramming in OSCC cells. By promoting fatty acid uptake and utilization, this pathway supports the heightened proliferative and invasive capacities of OSCC, contributing to tumor progression and aggressiveness. Given the crucial role of this pathway in OSCC development, targeting its components presents a novel therapeutic opportunity. ISL, a naturally occurring chalcone derived from traditional Chinese medicine, has garnered attention due to its multifaceted antitumor effects, including modulation of metabolic and signaling pathways [30,50]. ISL belongs to a broader class of plant-derived chalcones and flavonoids whose activity in oral malignancy has been reviewed elsewhere, with quercetin representing one of the most extensively profiled compounds modulating both metabolic and signalling pathways in oral cancer. Our data are consistent with a model in which ISL suppresses FABP5-mediated lipid metabolism and OSCC invasion in association with the circPOLB/miR-548ae-3p/c-MYC axis, and the partial rescue of FABP5 expression by miR-548ae-3p inhibition (Fig. 4E,F) places miR-548ae-3p within the path through which ISL acts. Regarding therapeutic potential, while our data on ISL are promising, the translational application faces challenges typical of natural chalcones, particularly regarding bioavailability and optimal dosing windows. Future clinical translation would require developing improved delivery systems (e.g., nanoparticle-based formulations) to enhance tumor accumulation.

Despite this study’s insights, several limitations exist. First, this study relies primarily on *in vitro* experiments without *in vivo* validation, requiring follow-up animal studies to confirm key effects. Second, although knockdown of circPOLB combined with miR-548ae-3p inhibition restores c-MYC expression (Fig. 2H,I) and miR-548ae-3p inhibition partially restores FABP5 expression under ISL treatment (Fig. 4E,F), direct genetic rescue at the downstream nodes—re-expression of c-MYC or a miRNA-resistant FABP5 in miR-548ae-3p-overexpressing cells, and FABP5 knockdown to phenocopy the lipid phenotype—was not performed; the link from miR-548ae-3p/c-MYC to the FABP5-associated metabolic phenotype therefore remains correlative and will require formal causal validation. Besides, our clinical inference relies on the TCGA-HNSC cohort, which encompasses head and neck squamous carcinomas more broadly than oral cavity tumors alone; circPOLB and miR-548ae-3p were not independently validated in OSCC patient specimens, and the c-MYC/FABP5 correlation, although statistically significant, is quantitatively modest, so targeted validation in OSCC-specific cohorts is required before the axis can be advanced as a prognostic biomarker. Finally, validation in HPV-positive or MYC-low OSCC backgrounds, and xenograft or orthotopic *in vivo* studies including pharmacokinetic and safety profiling of ISL, will be essential before the axis can be considered a tractable therapeutic target.

## 5 Conclusions

This study identifies miR-548ae-3p as a pivotal tumor suppressor in OSCC, demonstrating that it attenuates malignant proliferation, invasive capacity, and fatty acid metabolism via the circPOLB/miR-548ae-3p/c-MYC signaling axis. This regulatory effect is characterized by a significant downregulation of FABP5. Furthermore, pharmacological intervention targeting lipid metabolic reprogramming—specifically through natural small-molecule agents such as ISL—presents a promising therapeutic strategy for OSCC. These findings highlight a viable metabolic-targeted approach that warrants further translational and clinical investigation.

## Data Availability

The datasets and materials supporting the findings of this study are available from the corresponding authors upon reasonable request.
